# Supplementary material for: Sphingosine 1-phosphate mediates adiponectin receptor signaling essential for lipid homeostasis and embryogenesis
Source: Nat Commun. 2022 Nov 22;13:7162. doi: 10.1038/s41467-022-34931-0 (PMC9684441; doi:10.1038/s41467-022-34931-0)
Supplement: Supplementary file 1 — Supplementary Information [file 41467_2022_34931_MOESM1_ESM.pdf]

TITLE:

SPHINGOSINE 1-PHOSPHATE MEDIATES ADIPONECTIN RECEPTOR SIGNALING ESSENTIAL FOR LIPID HOMEOSTASIS AND EMBRYOGENESIS

RUNNING TITTLE: S1P promotes membrane homeostasis

AUTHORS: Mario Ruiz[1*], Ranjan Devkota[1], Dimitra Panagaki[1], Per-Olof Bergh[2], Delaney Kaper[1], Marcus Henricsson[2], Ali Nik[1], Kasparas Petkevicius[3], Johanna L. Höög[1], Mohammad Bohlooly-Y[3], Peter Carlsson[1], Jan Borén[2], Marc Pilon[1*]

AFFILIATIONS:
[1]Dept.Chemistry and Molecular Biology, Univ. Gothenburg, 405 30 Gothenburg, Sweden,
[2]Dept. Molecular and Clinical Medicine/Wallenberg Laboratory, Institute of Medicine, Univ. of Gothenburg, 414 67 Gothenburg, Sweden,
[3]Discovery Biology, Discovery Sciences, R&D, AstraZeneca, Gothenburg, Sweden.

CORRESPONDING TO: * Mario Ruiz (mario.ruiz.garcia@gu.se) or Marc Pilon (marc.pilon@cmb.gu.se), Dept. Chem Mol Biol, Univ. Gothenburg, Box 462, Gothenburg, SE-405 30, Sweden. Tel: +46 31 786 4952.

Supplementary Information

- Supplementary Fig. 1-8
- Supplementary Table 1-2
- Supplementary References
- Uncropped Blot S6D

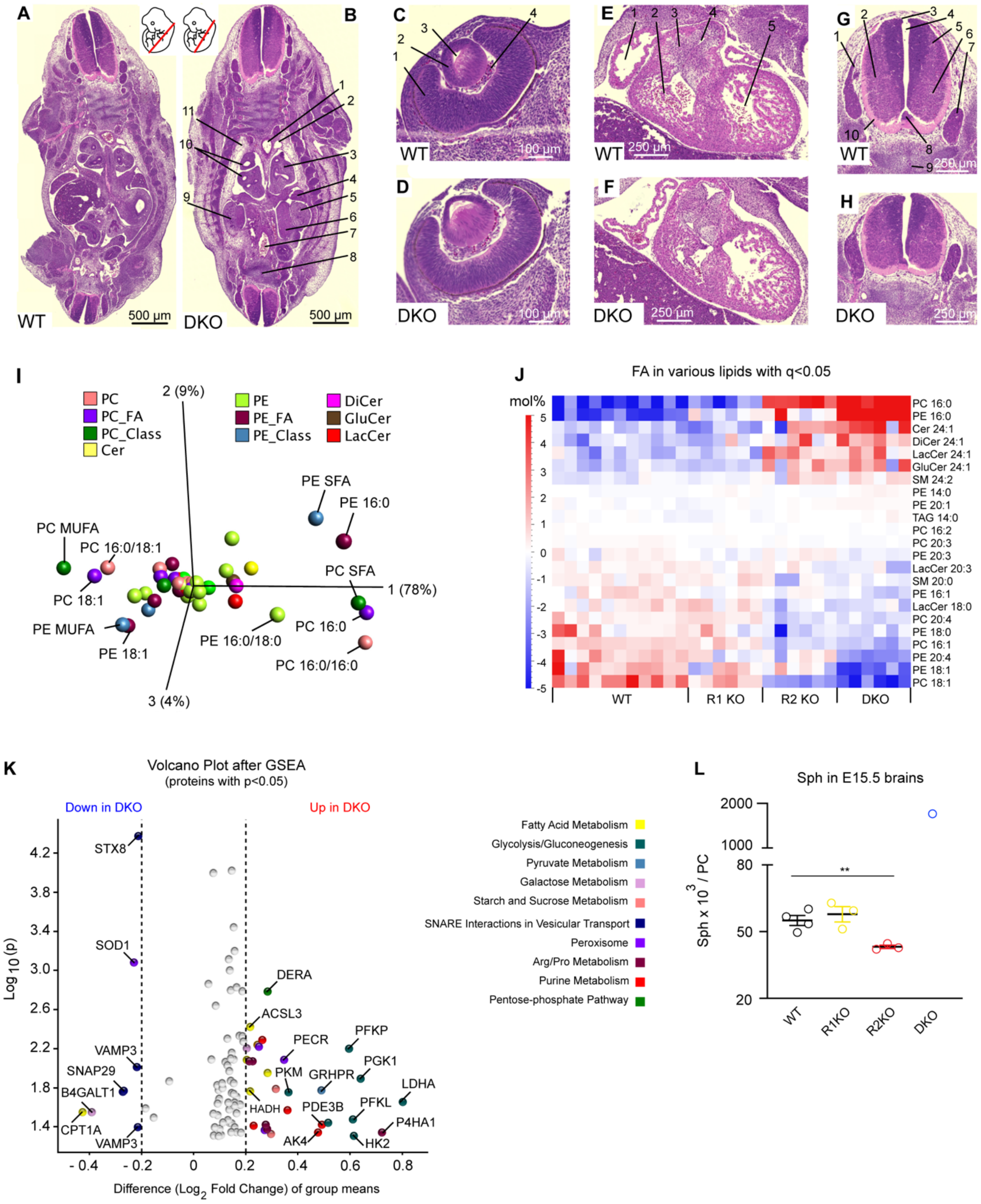
A
B
WT
DKO
500 µm
C
D
100 µm
E
F
250 µm
G
H
I
PC
PC_FA
PC_Class
Cer
PE
PE_FA
PE_Class
DiCer
GluCer
LacCer
2 (9%)
1 (78%)
3 (4%)
PE SFA
PE 16:0
PC MUFA
PC 16:0/18:1
PC SFA
PC 18:1
PE MUFA
PE 18:1
PE 16:0/18:0
PC 16:0
PC 16:0/16:0
J
FA in various lipids with q<0.05
mol%
PC 16:0
PE 16:0
Cer 24:1
DiCer 24:1
LacCer 24:1
GluCer 24:1
SM 24:2
PE 14:0
PE 20:1
TAG 14:0
PC 16:2
PC 20:3
PE 20:3
LacCer 20:3
SM 20:0
PE 16:1
LacCer 18:0
PC 20:4
PE 18:0
PC 16:1
PE 20:4
PE 18:1
PC 18:1
WT
R1 KO
R2 KO
DKO
K
Volcano Plot after GSEA
(proteins with p<0.05)
Down in DKO
Up in DKO
Log 10 (p)
Difference (Log2 Fold Change) of group means
STX8
SOD1
VAMP3
SNAP29
B4GALT1
CPT1A
DERA
ACSL3
PECR
PFKP
PGK1
PKM
GRHPR
LDHA
HADH
PDE3B
PFKL
P4HA1
AK4
HK2
Fatty Acid Metabolism
Glycolysis/Gluconeogenesis
Pyruvate Metabolism
Galactose Metabolism
Starch and Sucrose Metabolism
SNARE Interactions in Vesicular Transport
Peroxisome
Arg/Pro Metabolism
Purine Metabolism
Pentose-phosphate Pathway
L
Sph in E15.5 brains
Sph x 10^3 / PC
WT
R1KO
R2KO
DKO
**

Supplementary Fig.1 Membrane Lipid Composition Defects Precede Embryonic Lethality in DKO Mice (related to Fig.1).

(A-H) Sections of WT and DKO embryos at E12.5 stained with H&E. In C: 1. thoracic (descending) aorta; 2. left subcardinal vein; 3. segmental bronchus (left lung); 4. left lobe of liver; 5. left metanephros (kidney); 6. rete ovarii (early stage of differentiation); 7. sacral vein; 8. notochord; 9. right metanephros (kidney); 10. segmental bronchus (right lung); 11. loose connective tissue forming wall of pericardio-peritoneal cavity. In D: 1. inner (neural) layer of retina; 2. lens; 3. lens vesicle; 4. hyaloid cavity. In F: 1. right atrium; 2. right ventricle; 3. leaflets of pulmonary valve; 4. origin of pulmonary trunk; 5. left ventricle. In H: 1. dorsal nerve root; 2. mantle; 3. roof plate; 4. central canal; 5. alar plate; 6. basal plate; 7. dorsal root ganglion; 8. floor plate; 9. notochord; 10. marginal zone. Three embryos of each genotype were examined with similar results.

(I) PCA showing the variables responsible for the genotype separation in Fig.1D.

(J) Heatmap of all the lipid species with a $q<0.05$ when comparing WT, R1KO, R2KO, DKO embryos at E12.5. Variables considered as in panel D. Note that WT embryos were enriched in PC with 16:1 and PE with 18:1 (both MUFA) whereas DKO had elevated PC with 16:0 and PE with 16:0 (SFA). Related lipidomics are included in Supplementary Data 2.

(K) Volcano plot of the proteomics showing all significant proteins after GSEA (97 in total), between WT and DKO at E12.5. Note that only the proteins with a log2 difference > than 0.2 from WT and with a $p<0.05$ are colored and labeled. See Supplementary Data 3 for the full list.

(L) Sph abundance in mouse embryonal brains at E15.5. n=4, 3, 3 and 1 biologically independent replicate for WT, R1KO, R2KO and DKO, respectively. Error bars show the standard error of the mean, and t-tests (two sided and assuming normality) were used to identify significant differences. Related lipidomics are shown Supplementary Data S2.

See also Supplementary Data 1-3. Data are represented as mean ± SEM. $^{**}p<0.01$. Source data are provided as a Source Data file.

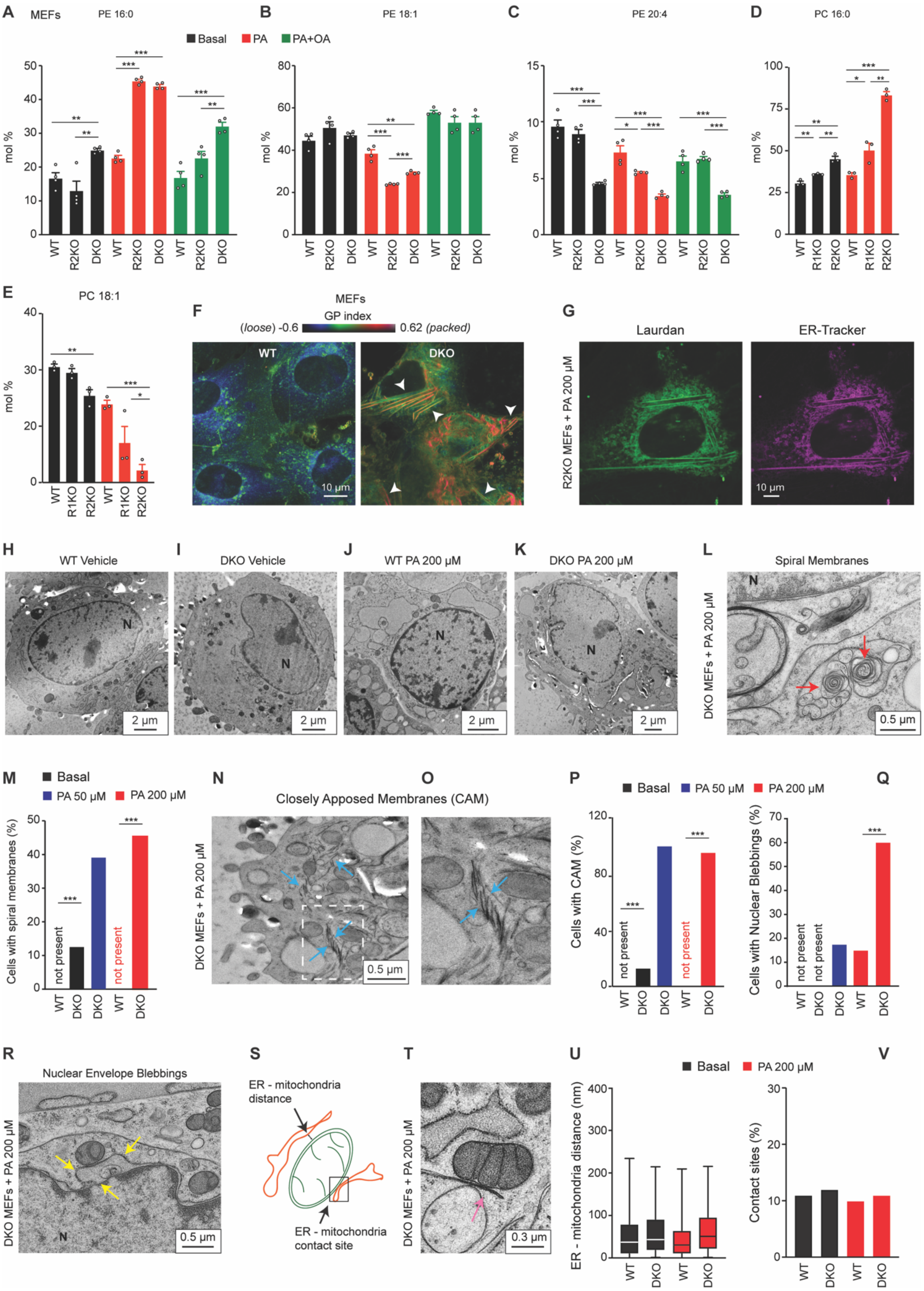
A
MEFs
PE 16:0
B
PE 18:1
C
PE 20:4
D
PC 16:0
Basal
PA
PA+OA
mol %
WT
R2KO
DKO
R1KO
E
PC 18:1
F
MEFs
GP index
(loose) -0.6
0.62 (packed)
WT
DKO
10 μm
G
Laurdan
ER-Tracker
R2KO MEFs + PA 200 μM
H
WT Vehicle
I
DKO Vehicle
J
WT PA 200 μM
K
DKO PA 200 μM
L
Spiral Membranes
DKO MEFs + PA 200 μM
N
2 μm
0.5 μm
M
Basal
PA 50 μM
PA 200 μM
Cells with spiral membranes (%)
not present
N
Closely Apposed Membranes (CAM)
O
P
Cells with CAM (%)
Q
Cells with Nuclear Blebbings (%)
R
Nuclear Envelope Blebbings
S
ER - mitochondria distance
ER - mitochondria contact site
T
0.3 μm
U
Basal
PA 200 μM
ER - mitochondria distance (nm)
V
Contact sites (%)

Supplementary Fig.2 S1P Rescues Membrane Homeostasis Defects Caused by the Absence of AdipoR2 in MEFs (related to Fig.2).

(A-E) PA (16:0), OA (18:1) and AA (20:4) abundance (mol%) in the PC and/or PE of WT, R1KO R2KO and DKO MEFs cultivated in the presence of vehicle, PA 200 μM ± OA 200 μM for 18 h. For A-C, n=4 independent biological replicates per condition. For D-E, n=3 independent biological replicates per condition. Related lipidomics are shown in Supplementary Data 4.

(F) Pseudocolor images of WT and DKO MEFs challenged with PA 200 μM and stained with Laurdan. Note that pixels with highly packed lipids are colored in red and appear only in DKO MEFs. White arrows indicate long and packed structures exclusively present in DKO cells. **(G)** Single channel confocal images of live R2KO MEFs stained with Laurdan (left picture) and ER-Tracker (right picture). Merged image is shown in Fig.2I.

(H-K) Electron microscopy pictures with a general cellular view of WT and DKO MEFs exposed to vehicle (F-G) and PA 200 μM (H-I).

(L-M) DKO MEFs images showing distinct spiral membranes (marked by red arrows) in the cytoplasm and the quantification. For each condition from left to right, n=27, 27, 24, 23 and 22. N in the pictures indicates the nucleus. **(N-P)** Representative image (and zoom in) of DKO MEFs exposed to PA 200 μM and showing electron dense closely apposed membranes (CAM, labeled with blue arrows) and the quantification (n=22-27 sections). The white square in N indicates the region magnified in O. **(Q-R)** Representative image of DKO MEFs treated with PA 200 μM and showing nuclear envelop blebbing (yellow arrows) and the quantification (n=17-27 sections). N in the pictures indicates the nuclei.

(S-V) Cartoon, representative EM image and quantification of ER-mitochondria distance (n≈100) and contact-sites in WT or DKO MEFs in basal media and treated with PA 200 μM. Error bars show the standard error of the mean, except for U where the boxes indicate the 25th to 75th percentile while the whiskers indicate the data points still within 1.5 of the box range. t-tests (two sided and assuming normality) were used to identify significant differences between treatments. *p<0.05, **p<0.01, ***p<0.001. See also Supplementary Data 4. Source data are provided as a Source Data file.

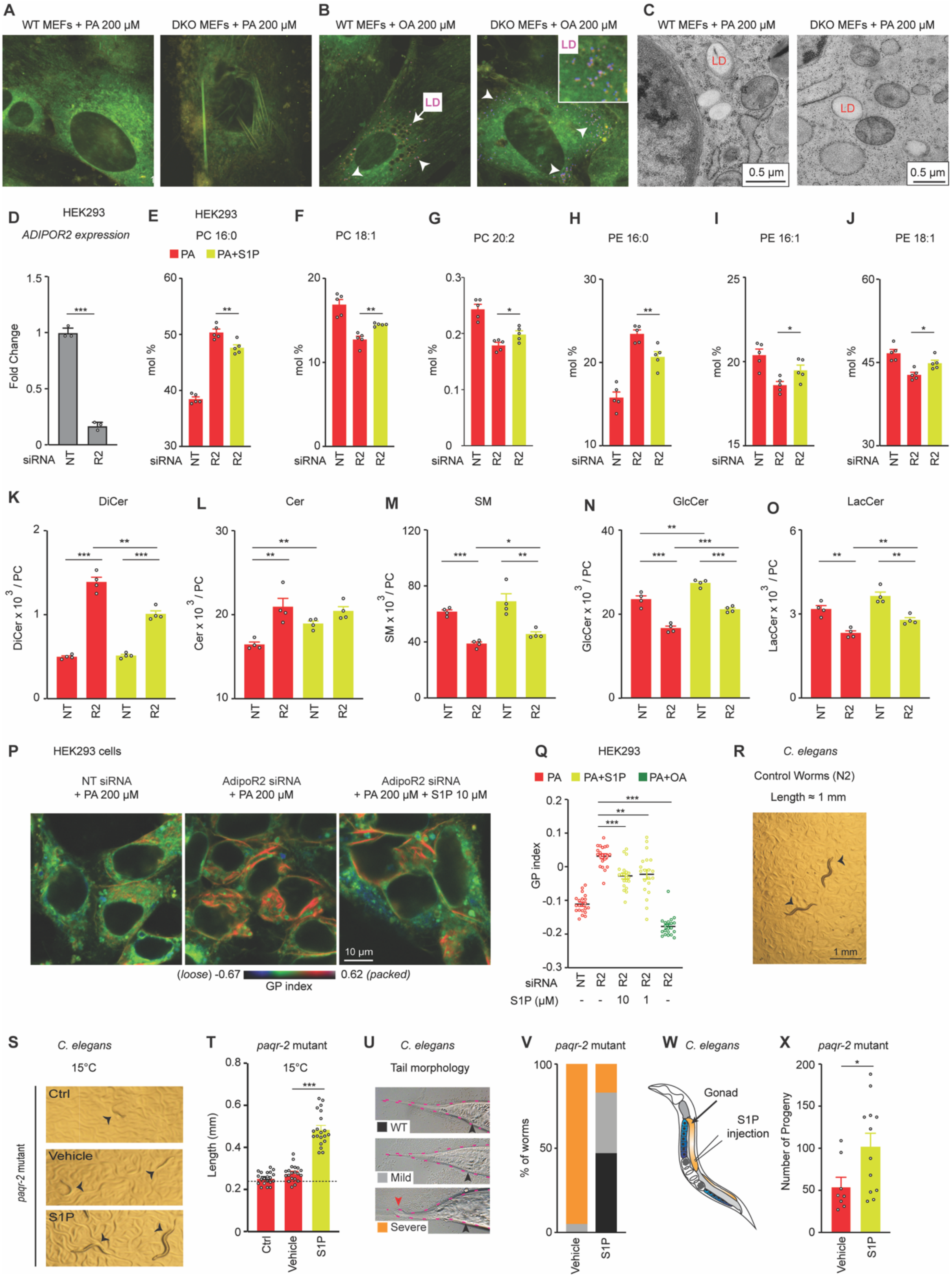

A
WT MEFs + PA 200 µM
DKO MEFs + PA 200 µM
B
WT MEFs + OA 200 µM
DKO MEFs + OA 200 µM
LD
C
WT MEFs + PA 200 µM
DKO MEFs + PA 200 µM
0.5 µm
D
HEK293
ADIPOR2 expression
Fold Change
siRNA NT R2
E
HEK293
PC 16:0
PA PA+S1P
mol %
F
PC 18:1
G
PC 20:2
H
PE 16:0
I
PE 16:1
J
PE 18:1
K
DiCer
DiCer x 10^3 / PC
L
Cer
Cer x 10^3 / PC
M
SM
SM x 10^3 / PC
N
GlcCer
GlcCer x 10^3 / PC
O
LacCer
LacCer x 10^3 / PC
P
HEK293 cells
NT siRNA + PA 200 µM
AdipoR2 siRNA + PA 200 µM
AdipoR2 siRNA + PA 200 µM + S1P 10 µM
10 µm
(loose) -0.67
0.62 (packed)
GP index
Q
HEK293
PA PA+S1P PA+OA
GP index
siRNA NT R2 R2 R2 R2
S1P (µM) - - 10 1 -
R
C. elegans
Control Worms (N2)
Length ≈ 1 mm
1 mm
S
C. elegans
15°C
paqr-2 mutant
Ctrl
Vehicle
S1P
T
paqr-2 mutant
15°C
Length (mm)
Ctrl Vehicle S1P
U
C. elegans
Tail morphology
WT
Mild
Severe
V
paqr-2 mutant
% of worms
Vehicle S1P
W
C. elegans
Gonad
S1P injection
X
paqr-2 mutant
Number of Progeny
Vehicle S1P

Supplementary Fig.3 Loss of AdipoRs Causes Multiple Membrane Defects in MEFs, HEK293 Cells and in *C. elegans* (related to Fig.2)**.**
(A-B) Representative images of MEFs stained with Laurdan (green and red color) and LipidSpot 610 (purple dots pointed by white arrows).
(C) Representative EM images of MEFs.
(D) qPCR showing the efficiency of the AdipoR2 knockdown in HEK293 cells. n=3 technical replicate per condition.
(E-J) PA 16:0), OA (18:1) and eicosadienoic (20:2n-6) fatty acid abundance (mol%) in the PC and PA, PalOA (16:1) and OA in the PE of HEK293 cells cultivated in the presence of PA 200 µM ± S1P 1 µM and treated with NT and AdipoR2 siRNA. n=5 independent biological replicates per condition. Related Lipidomics are shown in Supplementary Data 6.
(K-O) DiCer, Cer, SM, GlcCer and LacCer in HEK293 cells cultivated in the presence of PA 200 µM ± S1P 1 µM and treated with NT or AdipoR2 siRNA. n=4 independent biological replicates per condition. From Supplementary Data 6.
(P-Q) Pseudocolor images and GP index of HEK293 cells treated with PA 200 µM ± S1P (10 and 1 µM) or ± OA 200 µM and stained with Laurdan dye.
(R) Representative picture of WT 1-day adult *C. elegans*.
(S-T) Representative pictures and quantification of the length of *paqr*-2 mutant worms (arrowheads) grown on control plates or vehicle ± S1P 25 µM at 15°C for 144 h. The dashed line in S represents the approximate length of the L1s at the start of the experiments. n= 20 worms measured per condition.
(U-V) Representative images and quantification (n=50 worms/genotype) of the tail tip morphology of 1-day adult *C. elegans*: WT phenotype, and mild and severe defects are shown. Shorter and broader tail tips were scored as mild defect, whereas deformed and bumpy tails were scored as severe defect (indicated by the red arrow). Black arrows indicate the anus.
(W-X) Schematic representation of *C. elegans*. The region of the gonad where S1P was injected is highlighted in orange. Brood size of *paqr-2* mutant injected with vehicle ± S1P 10 µM. n=8 and 12 independently injected worms for vehicle the S1P treatment, respectively.
Data are means ± SEM, except in D: ± SD. t-tests (two sided and assuming normality) were used to identify significant differences between treatments. *p<0.05, **p<0.01, ***p<0.001. See also Supplementary Data 6. Source data are provided as a Source Data file.

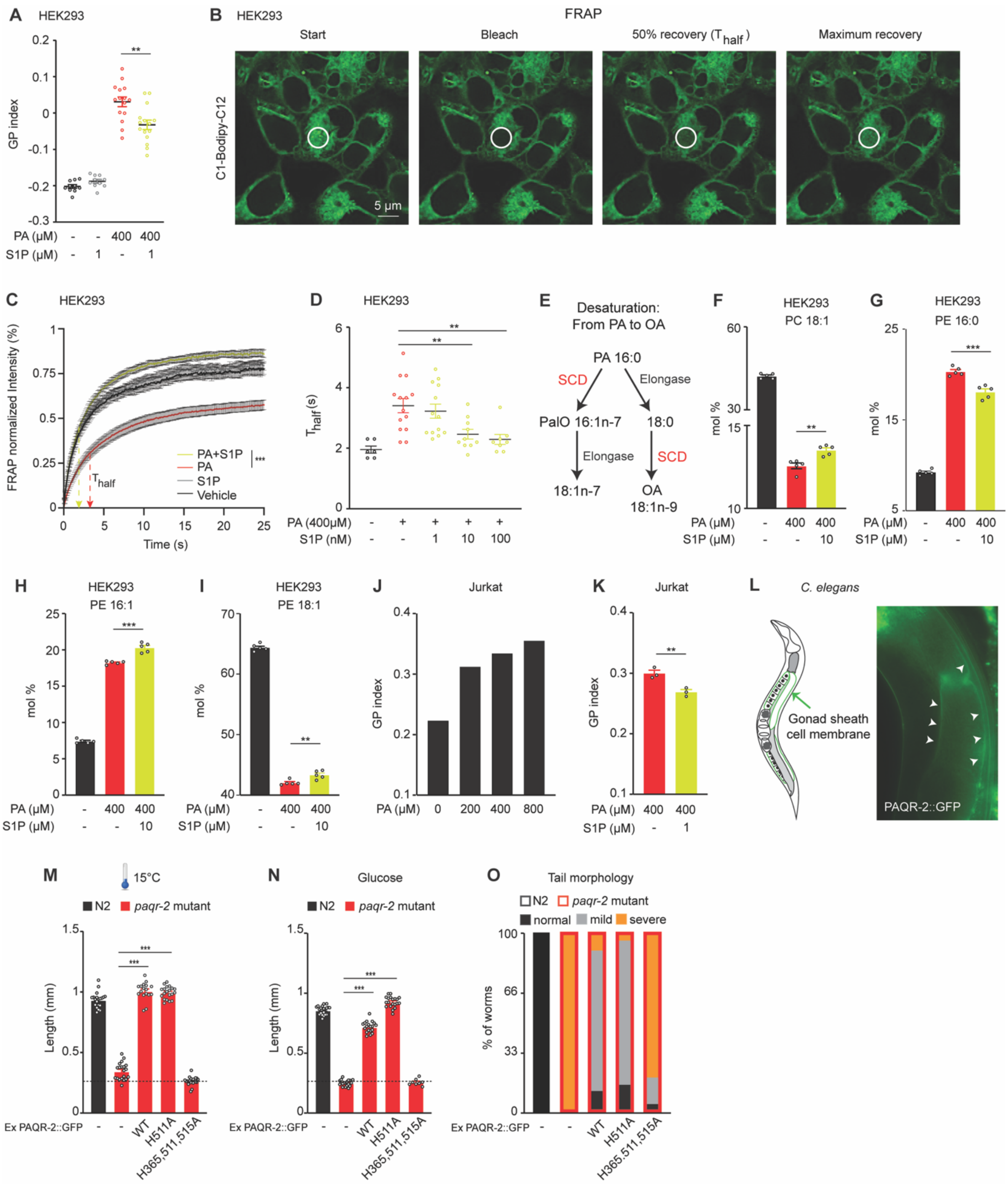

A HEK293
GP index
PA (μM) - - 400 400
S1P (μM) - 1 - 1
B HEK293
FRAP
Start
Bleach
50% recovery (T half)
Maximum recovery
C1-Bodipy-C12
5 μm
C HEK293
FRAP normalized Intensity (%)
PA+S1P
PA
S1P
Vehicle
T half
Time (s)
D HEK293
T half (s)
PA (400μM) - + + + +
S1P (nM) - - 1 10 100
E
Desaturation:
From PA to OA
PA 16:0
SCD
Elongase
PalO 16:1n-7
18:0
Elongase
SCD
18:1n-7
OA
18:1n-9
F HEK293
PC 18:1
mol %
PA (μM) - 400 400
S1P (μM) - - 10
G HEK293
PE 16:0
mol %
PA (μM) - 400 400
S1P (μM) - - 10
H HEK293
PE 16:1
mol %
PA (μM) - 400 400
S1P (μM) - - 10
I HEK293
PE 18:1
mol %
PA (μM) - 400 400
S1P (μM) - - 10
J Jurkat
GP index
PA (μM) 0 200 400 800
K Jurkat
GP index
PA (μM) 400 400
S1P (μM) - 1
L C. elegans
Gonad sheath cell membrane
PAQR-2::GFP
M 15°C
N2 paqr-2 mutant
Length (mm)
Ex PAQR-2::GFP - - WT H511A H365,511,515A
N Glucose
N2 paqr-2 mutant
Length (mm)
Ex PAQR-2::GFP - - WT H511A H365,511,515A
O Tail morphology
N2 paqr-2 mutant
normal mild severe
% of worms
Ex PAQR-2::GFP - - WT H511A H365,511,515A

Supplementary Fig.4. S1P Promotes Membrane Homeostasis in Multiple Human Cell Types and in *C. elegans* (related to Fig.3).

(A) Average GP index from several images of HEK293 cells treated as indicated. For each condition, from left to right, n=10, 10, 15 and 15 separate images analyzed.

(B-D) C1-Bodipy-C12 staining of live HEK293 cells indicating different phases during a FRAP experiment (start, bleach, 50% recovery and end). The white circle represents the FRAP region. Panel C shows the normalized fluorescence intensity during the FRAP experiment of cells treated with vehicle, S1P 1 µM, and PA 400 µM ± S1P 1 µM. Panel D shows the average Thalf values from the curves in C. In D, for each condition, from left to right, n=6, 14, 13, 10 and 7 separate images analyzed.

(E) Pathway showing how PA (16:0) is desaturated (and elongated) to become 18:1. **(F-I)** PalO (16:1) and OA (18:1) abundance (mol%) in the PC and PE of HEK293 cells treated as indicated. In G-I, n=5 independent biological replicates per condition. Data from Supplementary Data S5.

(J-K) Average GP index from cytometry experiments of Jurkat E6.1 cells treated with PA (panel J) or with PA 400 µM ± S1P (panel K) for 18 h and stained with Laurdan. In J, n=1 and K, n=3 independent biological replicates analyzed by flow cytometry.

(L) Representation of *C. elegans* highlighting the gonad sheath cell membrane in green (tissue were PAQR-2::GFP is easily visualized) and a fluorescent picture of the actual gonad sheath cell membrane (white arrows).

(M-N) Length of N2 and *paqr-2* mutant worms expressing PAQR-2::GFP with WT sequence or with H511A and H365,511,516A variants. Worms were grown at 15°C for 144 h (panel M) or at 20°C on glucose plates for 72 h (panel N). Note that glucose is readily converted to SFAs by the dietary *E. coli*(Devkota et al., 2017). The dashed line represents the approximate length of the L1s at the start of the experiments. In M, from left to right, n= 20, 20, 15, 20 and 20 worms measured per condition. In N, from left to right, n=20, 19, 19, 20 and 8 worms measured per condition.

(O) Quantification of the tail tip morphology of 1-day adult N2 and *paqr-2* mutant worms expressing different PAQR-2::GFP variants (n=50 worms/genotype DK). Examples of the different tail morphologies are shown in Supplementary Fig.3U.

Data are means ± SEM, and t-tests (two sided and assuming normality) were used to identify significant differences between treatments. Data shows mean ± SEM. *p<0.05, **p<0.01, ***p<0.001. See also Supplementary Data 5-S6. Source data are provided as a Source Data file.

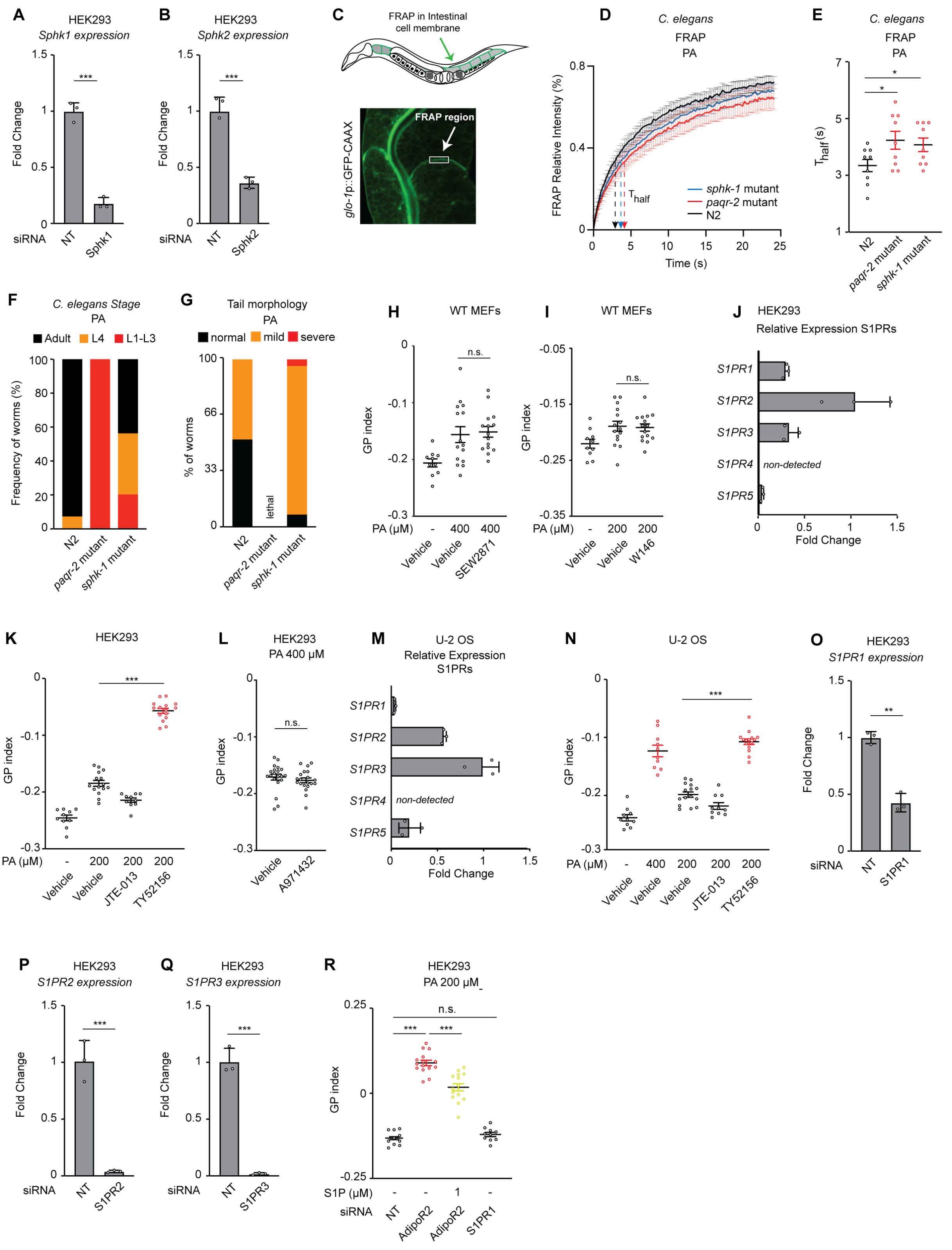
A
HEK293
Sphk1 expression
Fold Change
siRNA
NT
Sphk1
***
B
HEK293
Sphk2 expression
Fold Change
siRNA
NT
Sphk2
***
C
FRAP in Intestinal cell membrane
glo-1p::GFP-CAAX
FRAP region
D
C. elegans
FRAP
PA
FRAP Relative Intensity (%)
Time (s)
Thalf
sphk-1 mutant
paqr-2 mutant
N2
E
C. elegans
FRAP
PA
Thalf (s)
N2
paqr-2 mutant
sphk-1 mutant
F
C. elegans Stage
PA
Adult
L4
L1-L3
Frequency of worms (%)
N2
paqr-2 mutant
sphk-1 mutant
G
Tail morphology
PA
normal
mild
severe
% of worms
lethal
N2
paqr-2 mutant
sphk-1 mutant
H
WT MEFs
GP index
n.s.
PA (μM)
Vehicle
Vehicle
SEW2871
I
WT MEFs
GP index
n.s.
PA (μM)
Vehicle
Vehicle
W146
J
HEK293
Relative Expression S1PRs
S1PR1
S1PR2
S1PR3
S1PR4
non-detected
S1PR5
Fold Change
K
HEK293
GP index
***
PA (μM)
Vehicle
Vehicle
JTE-013
TY52156
L
HEK293
PA 400 μM
GP index
n.s.
Vehicle
A971432
M
U-2 OS
Relative Expression S1PRs
S1PR1
S1PR2
S1PR3
S1PR4
non-detected
S1PR5
Fold Change
N
U-2 OS
GP index
***
PA (μM)
Vehicle
Vehicle
Vehicle
JTE-013
TY52156
O
HEK293
S1PR1 expression
Fold Change
**
siRNA
NT
S1PR1
P
HEK293
S1PR2 expression
Fold Change
***
siRNA
NT
S1PR2
Q
HEK293
S1PR3 expression
Fold Change
***
siRNA
NT
S1PR3
R
HEK293
PA 200 μM
GP index
n.s.
***
***
S1P (μM)
siRNA
NT
AdipoR2
AdipoR2
S1PR1

Supplementary Fig.5 Sphingosine Kinases and S1PR3 Are Required to Maintain Membrane Homeostasis in Mammalian Cells and *C. elegans* (related to Fig.4)

(A-B) qPCR showing the efficiencies of the Sphk1 and Sphk2 knockdowns. n=3 technical replicate per condition.

(C) Schematic representation of *C. elegans* (note that the membrane of intestinal cells is decorated in green representing a membrane-bound prenylated GFP) and a confocal image of the actual GFP signal in intestinal cells of *C. elegans*. The white square represents the FRAP region.

(D-E) Curves and T_{half} values of the normalized fluorescence during a FRAP experiment of worms grown in PA 2 mM plates. In E, n=9 worms scored for each genotype.

(F-G) Quantification of *C. elegans* larval stages and adulthood after 72 h growing at 20°C in F, and tail tip morphology of 1-day adult worms grown on plates with PA-loaded OP50 bacteria in G (n=50 worms/genotype). Examples of the different tail morphologies are shown in Supplementary Fig.3U.

(H-I) Average GP index from several images of WT MEFs treated with vehicle, PA 400 µM ± SEW2871 1 µM (S1PR1 agonist) in H and PA 400 µM ± W146 5 µM (S1PR1 antagonist) in I. In H, for each condition from left to right, n=10, 15 and 15 separate images analyzed. In I, for each condition from left to right, n=10, 15 and 15 separate images analyzed.

(J) Relative expression of S1PRs in HEK293 cells measured by qPCR. n=3 technical replicate per condition.

(K-L) Average GP index from several images of HEK293 cells treated with vehicle, PA 200 µM ± JTE-013 5 µM (S1PR2 antagonist), ± TY52156 5 µM (S1PR3 antagonist) in K and PA 400 µM ± A971432 1 µM (S1PR5 agonist) in L. In K, for each condition from left to right, n=10, 15, 10 and 15 separate images analyzed. In L, n=20 separate images analyzed per condition.

(M) Relative expression of S1PRs in U-2 OS cells measured by qPCR. n=3 technical replicate per condition.

(N) Average GP index from several images of U-2 OS cells treated with vehicle, PA 200 µM ± JTE-013 5 µM (S1PR2 antagonist), ± TY52156 5 µM (S1PR3 antagonist). For each condition from left to right, n=10, 10, 15, 10 and 15 separate images analyzed.

(O-Q) qPCR showing the efficiencies of the S1PR1, S1PR2, S1PR3 knockdowns. n=3 technical replicate per condition.

(R) Average GP index from several images of NT, AdipoR2 and S1PR1 siRNA HEK293 cells treated with PA 200 µM ± S1P 1 µM. For each condition from left to right, n=10, 15, 15 and 10 separate images analyzed.

Data shows mean ± SEM in D-E, H-I, K-L, N and R and ± SD in A-B, J, M and O-Q. t-tests (two sided and assuming normality) were used to identify significant differences between treatments. $*p<0.05$, $**p<0.01$, $***p<0.001$. Source data are provided as a Source Data file.

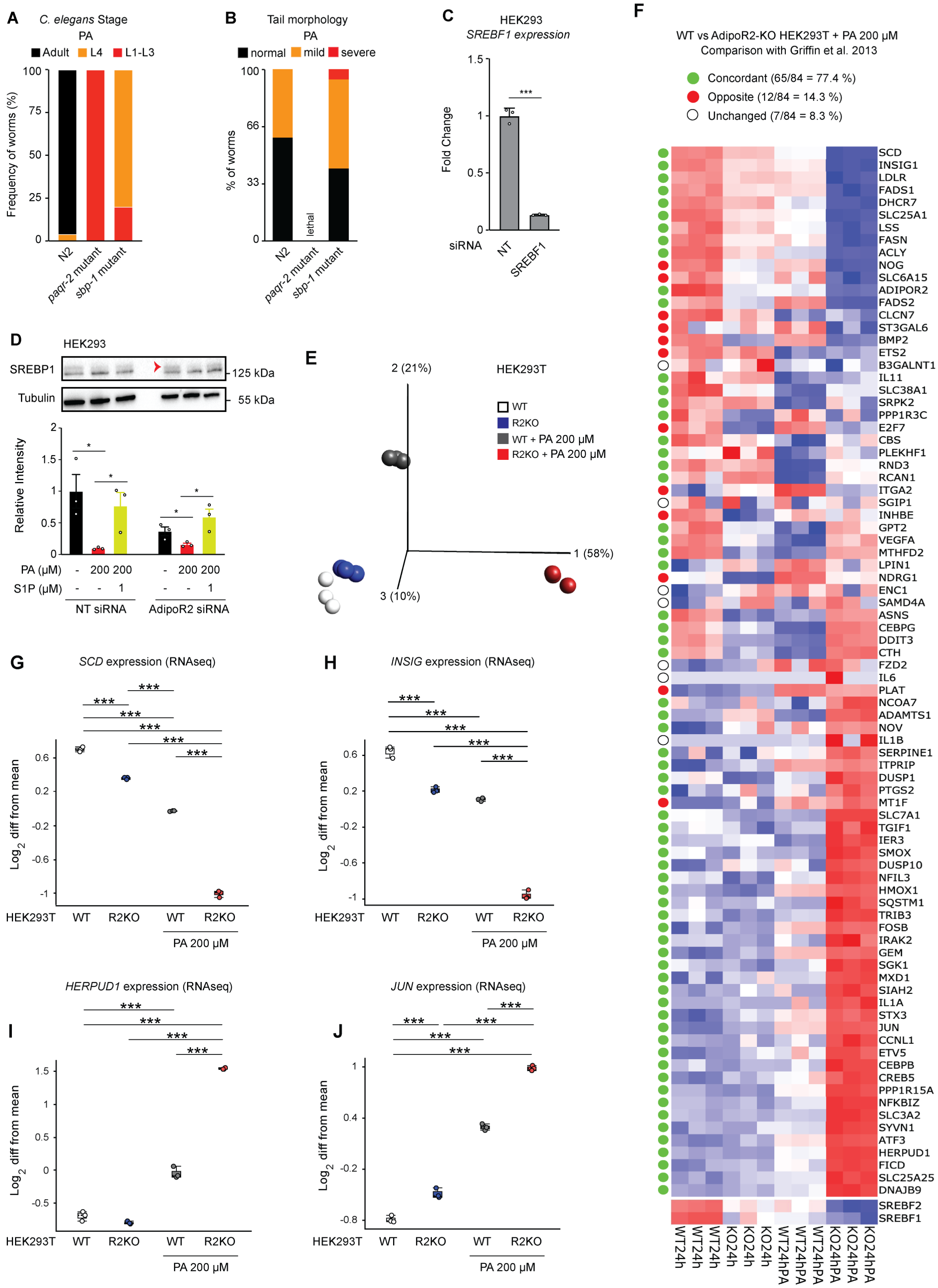
A
C. elegans Stage
PA
Adult L4 L1-L3
Frequency of worms (%)
N2
paqr-2 mutant
sbp-1 mutant
B
Tail morphology
PA
normal mild severe
% of worms
lethal
N2
paqr-2 mutant
sbp-1 mutant
C
HEK293
SREBF1 expression
Fold Change
siRNA
NT
SREBF1
D
HEK293
SREBP1
Tubulin
125 kDa
55 kDa
Relative Intensity
PA (µM)
S1P (µM)
NT siRNA
AdipoR2 siRNA
E
2 (21%)
1 (58%)
3 (10%)
HEK293T
WT
R2KO
WT + PA 200 µM
R2KO + PA 200 µM
F
WT vs AdipoR2-KO HEK293T + PA 200 µM
Comparison with Griffin et al. 2013
Concordant (65/84 = 77.4 %)
Opposite (12/84 = 14.3 %)
Unchanged (7/84 = 8.3 %)
SCD INSIG1 LDLR FADS1 DHCR7 SLC25A1 LSS FASN ACLY NOG SLC6A15 ADIPOR2 FADS2 CLCN7 ST3GAL6 BMP2 ETS2 B3GALNT1 IL11 SLC38A1 SRPK2 PPP1R3C E2F7 CBS PLEKHF1 RND3 RCAN1 ITGA2 SGIP1 INHBE GPT2 VEGFA MTHFD2 LPIN1 NDRG1 ENC1 SAMD4A ASNS CEBPG DDIT3 CTH FZD2 IL6 PLAT NCOA7 ADAMTS1 NOV IL1B SERPINE1 ITPRIP DUSP1 PTGS2 MT1F SLC7A1 TGIF1 IER3 SMOX DUSP10 NFIL3 HMOX1 SQSTM1 TRIB3 FOSB IRAK2 GEM SGK1 MXD1 SIAH2 IL1A STX3 JUN CCNL1 ETV5 CEBPB CREB5 PPP1R15A NFKBIZ SLC3A2 SYVN1 ATF3 HERPUD1 FICD SLC25A25 DNAJB9
SREBF2 SREBF1
WT24h WT24h WT24h KO24h KO24h KO24h WT24hPA WT24hPA WT24hPA KO24hPA KO24hPA KO24hPA
G
SCD expression (RNAseq)
H
INSIG expression (RNAseq)
I
HERPUD1 expression (RNAseq)
J
JUN expression (RNAseq)
Log2 diff from mean
HEK293T WT R2KO WT R2KO
PA 200 µM

Supplementary Fig.6. S1P Signaling Via SREBP1 Maintains Membrane Homeostasis in Human Cells and *C. elegans* (related to Fig.5).

(A-B) Quantification of *C. elegans* larval stages and adulthood in N2, *paqr-2* and *sbp-1* mutants after 72 h growing at 20°C in A and Tail tip morphology of 1-day adult worms grown on plates with PA-loaded OP50 bacteria in B (n=50 worms/genotype). Examples of the different tail morphologies are shown in Fig.S3U.

(C) qPCR result showing the efficiency of the SREBF1 siRNA knockdown by siRNA in HEK293 cells. n=3 technical replicate per condition.

(D) Western-Blot and quantification (n=3 experiments) of NT and AdipoR2 siRNA HEK293 cells treated with vehicle and PA 200 µM ± S1P 1 µM. The red arrow points to the precursor SREBP1 band. Note the presence of an unspecific band just below SREBP1. Uncropped blots in Source Data.

(E) PCA plot based on 84 SREBP-regulated genes from [1] of WT or AdipoR2-KO HEK293T cells in basal media or basal media supplemented with PA 200 µM for 24 h. Note that genotypes are well separated upon PA treatment.

(F) All 84 genes from [1] listed in the order of the PC1, and labelled as to whether their changes in expression matched [1]. *SREBF1/2* were both downregulated in AdipoR2-KO cells and were added at the bottom of the heat map.

(G-J) Plots of four genes whose expression its highly altered by the loss of AdipoR2 or SREBP1/2. n=3 independent biological replicates per condition.

Data are represented as mean ± SEM in D and ± SD in C and G-J. t-tests (two sided and assuming normality) were used to identify significant differences between treatments. *$p<0.05$, ***$p<0.001$. See also Supplementary Data 5. Source data are provided as a Source Data file.

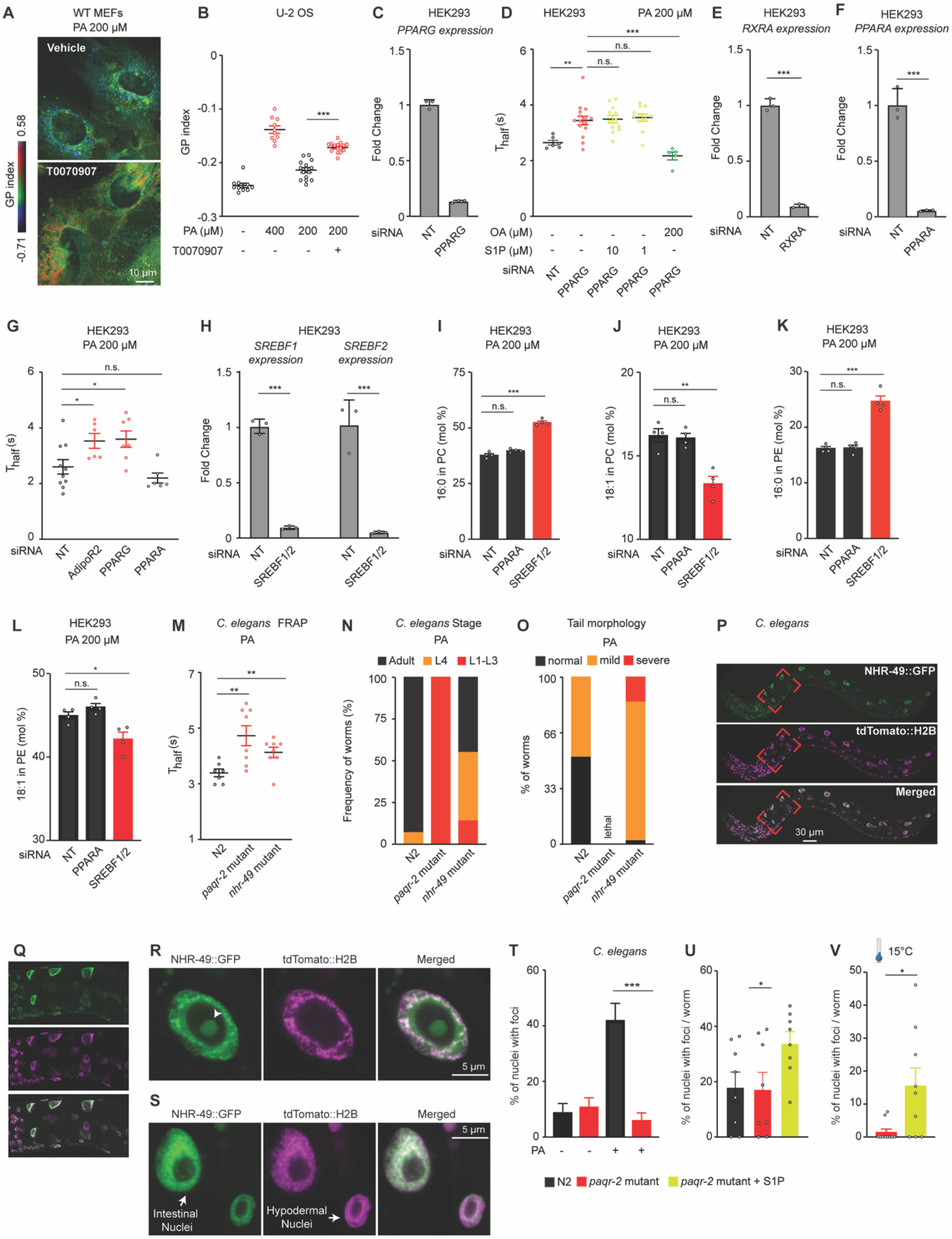

A
WT MEFs
PA 200 µM
Vehicle
T0070907
GP index
0.58
-0.71
10 µm
B
U-2 OS
GP index
PA (µM)
T0070907
C
HEK293
PPARG expression
Fold Change
siRNA
NT
PPARG
D
HEK293
PA 200 µM
Thalf (s)
OA (µM)
S1P (µM)
n.s.
E
HEK293
RXRA expression
RXRA
F
HEK293
PPARA expression
PPARA
G
AdipoR2
H
SREBF1 expression
SREBF2 expression
SREBF1/2
I
16:0 in PC (mol %)
J
18:1 in PC (mol %)
K
16:0 in PE (mol %)
L
18:1 in PE (mol %)
M
C. elegans FRAP
PA
N2
paqr-2 mutant
nhr-49 mutant
N
C. elegans Stage
Adult
L4
L1-L3
Frequency of worms (%)
O
Tail morphology
normal
mild
severe
% of worms
lethal
P
C. elegans
NHR-49::GFP
tdTomato::H2B
Merged
30 µm
Q
R
5 µm
S
Intestinal Nuclei
Hypodermal Nuclei
T
% of nuclei with foci
U
% of nuclei with foci / worm
V
15°C
N2
paqr-2 mutant
paqr-2 mutant + S1P

Supplementary Fig.7. S1P Signaling Via PPARγ Maintains Membrane Homeostasis in Human/Mouse Cells and *C. elegans* (related to Fig.5).

(A) Pseudocolor images of WT MEFs treated with vehicle, PA 200 μM ± T0070907 1 μM (PPARγ antagonist). Quantification of several images is shown in Fig.5D.

(B) Average GP index of U-2 OS cells treated with vehicle, PA 400 μM and PA 200 μM ± T0070907 1 μM. For each condition from left to right, n=10, 10, 15 and 15 separate images analyzed.

(C) qPCR showing the efficiency of the PPARG knockdown. n=3 technical replicates per condition.

(D) FRAP experiment of NT and PPARG siRNA HEK293 cells treated with PA 200 μM ± S1P (10 or 1 μM) and ± OA 200 μM. For each condition from left to right, n=6, 15, 12, 11 and 5 separate cells were analyzed.

(E) qPCR showing the efficiency of the RXRA knockdown. n=3 technical replicates per condition.

(F) qPCR showing the efficiency of the PPARA knockdown. n=3 technical replicates per condition.

(G) FRAP experiment in HEK293 cells. For each condition from left to right, n=10, 6, 15, 7 and 6 separate cells were analyzed.

(H) qPCR showing the efficiency of the SREBF1+2 knockdown. n=technical replicates per condition.

(I-L) PA 16:0) and OA (18:1) abundance (mol%) in the PC and in the PE of HEK293 cells cultivated as indicated. n=4 independent biological replicates per condition. From Supplementary Data S6.

(M) FRAP experiment on N2, *paqr-2* and *nhr-49* mutants fed PA-loaded OP50 bacteria. For each genotype from left to right, n=7, 8, and 8 separate worms analyzed.

(N-O) Quantification of *C. elegans* larval stages and adulthood in N2, *paqr-2* and *nhr-49* mutants after 72 h growing at 20°C in N. Tail tip morphology of 1-day adult worms grown on plates with PA-loaded OP50 bacteria in O (n=50 worms/genotype). Examples of the different tail morphologies are shown in Fig.S3U.

(P-S) Images of *C. elegans* expressing NHR-49::GFP and tdTomato::H2B (nuclear marker). Panels Q shows zoom-in of P. R shows higher magnification of an intestinal nucleus with nucleolar foci (arrowhead) while S shows intestinal and hypodermal nuclei.

(T-V) Quantification of the presence of nucleolar foci in intestinal nuclei of N2 and *paqr-2* mutants grown on control plates (S) and on plates supplemented with S1P at 20°C (T) and 15°C (V). In T, from left to right, n=59, 52, 53 and 43 worms scored. In U, n=8 worms scored. In V, n=10 for *paqr-2* mutant and n=9 for *paqr-2* mutant + S1P.

Data shows mean ± SEM in B, D, G, I-L, S-U and ± SD in C, E-F, H. t-tests (two sided and assuming normality) were used to identify significant differences between treatments. *p<0.05, **p<0.01, ***p<0.001. See also Supplementary Data 5-6. Source data are provided as a Source Data file.

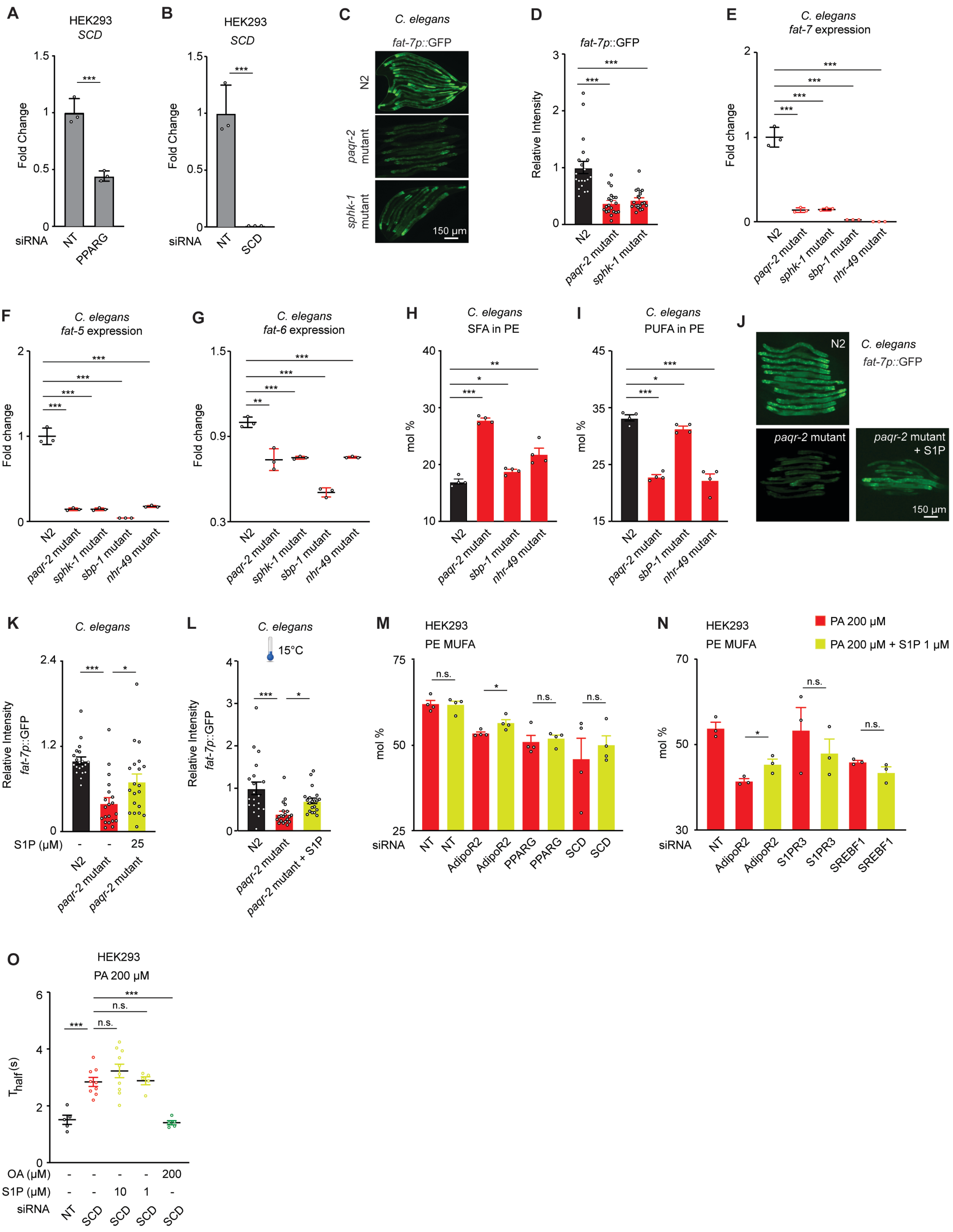

A
HEK293
SCD
Fold Change
siRNA
NT
PPARG
B
HEK293
SCD
C
C. elegans
fat-7p::GFP
N2
paqr-2 mutant
sphk-1 mutant
150 µm
D
fat-7p::GFP
Relative Intensity
E
C. elegans
fat-7 expression
Fold change
sbp-1 mutant
nhr-49 mutant
F
C. elegans
fat-5 expression
G
C. elegans
fat-6 expression
H
C. elegans
SFA in PE
mol %
I
C. elegans
PUFA in PE
J
C. elegans
fat-7p::GFP
paqr-2 mutant + S1P
K
C. elegans
Relative Intensity fat-7p::GFP
S1P (µM)
L
C. elegans
15°C
paqr-2 mutant + S1P
M
HEK293
PE MUFA
AdipoR2
SCD
N
HEK293
PE MUFA
PA 200 µM
PA 200 µM + S1P 1 µM
S1PR3
SREBF1
O
HEK293
PA 200 µM
T_{half} (s)
OA (µM)
S1P (µM)
n.s.

Figure S8. Role of SCD in Maintenance of Membrane Homeostasis in Human Cells and in *C. elegans* (related to Fig.6).
(A) Relative expression of *SCD* in NT and PPARG sRNA HEK293 cells measured by qPCR.
(B) qPCR result showing the efficiency of the SCD knockdown by siRNA in HEK293 cells n=3 technical replicates per condition.
(C-D) Representative images of *C. elegans* expressing GFP under the control of the *fat-7* promoter (*fat-7p*::GFP) and quantification of the fluorescence. N2 and *paqr-2* and *sphk-1* mutant worms were grown on control plates for 72 h.
(E-G) Relative expression of *fat-7, fat-5 and fat-6* in N2, *paqr-2*, *sphk1* and *nhr-49* mutant worms measured by qPCR.
(H-I) PA 16:0) and OA (18:1) abundance (mol%) and in the PE of *C. elegans* grown on PA 2 mM plates. Related lipidomics are shown in Supplementary Data 7.
(J-K) Representative images *C. elegans* expressing GFP under the control of the *fat-7* promoter (*fat-7p*::GFP) and the quantification of the fluorescence. N2 and *paqr-2* mutant worms were grown on control plates and plates supplemented with S1P 25 µM for 72 h.
(L) Quantification of the fluorescence of *C. elegans* expressing GFP under the control of the *fat-7* promoter (*fat-7p*::GFP) grown at 15°C on control plates and plates supplemented with S1P 25 µM for 144 h.
(M-N) MUFA abundance (mol%) and in the PE of HEK293 cells treated with different siRNA and PA 200 µM ± S1P 1 µM. Related lipidomics are shown in Fig.6C-F and in Supplementary Data 6.
(O) FRAP experiment of NT and SCD siRNA HEK293 cells treated with PA 200 µM ± S1P (10 or 1 µM) and ± OA 200 µM.
Data are represented as mean ± SEM in D-I, K-O, I and ± SD in A-B. t-tests (two sided and assuming normality) were used to identify significant differences between treatments. *p<0.05, **p<0.01, ***p<0.001. See also Supplementary Data 5-7. Source data are provided as a Source Data file.

SUPPLEMENTARY TABLE 1

PCR PRIMERS
Primers used in this study (some primers were designed for previous studies).

Sequence (5'-->3')	Organism	Purpose - Target	Reference
AGGCAGGGTAAGCTGATTAGCTATG<br>TCCACTGTGTCAGCTTCTCTGTTAC<br>GGGTGGGATTAGATAAATGCCTGCTCT	*Mus musculus*	Genotyping - *Adipor1*	2
GACGGAGTTTGTATGTGGTAGCGTC<br>TCTCTGCCTTTCCTTTTCATGGCTC<br>GGGCCAGCTCATTCCTCCCACTCAT	*Mus musculus*	Genotyping / *Adipor2*	2
CTGCCTTTCACCTTGGAGAC<br>CGTTTCCTGGGGATGAGATA	*Mus musculus*	qPCR - *Ddit3*	3
CATGGTTCTCACTAAAATGAAAGG<br>GCTGGTACAGTAACAACTG	*Mus musculus*	qPCR - *Hspa5*	3
ATGGCCGGCTATGGATGAT<br>CGAAGTCAAACTCTTTCAGATCCATT	*Mus musculus*	qPCR - *Atf4*	3
GAGTCCGCAGCAGGTG<br>TAAGACTCCTGCCTGACTGC	*Mus musculus*	qPCR - *sXBP1*	4
ACTACACAACGGGAGCAACAG<br>GATGGAAAGCAGGAGCAGAG	*Mus musculus*	qPCR - *S1pr1*	5
CTCACTGCTCAATCCTGTCATC<br>TTCACATTTTCCCTTCAGACC	*Mus musculus*	qPCR - *S1pr2*	5
TTCCCGACTGCTCTACCATC<br>CCAACAGGCAATGAACACAC	*Mus musculus*	qPCR - *S1pr3*	5
TGCGGGTGGCTGAGAGTG<br>TAGGATCAGGGCGAAGACC	*Mus musculus*	qPCR - *S1pr4*	5
CTTAGGACGCCTGGAAACC<br>CCCGCACCTGACAGTAAATC	*Mus musculus*	qPCR - *S1pr5*	5
GTCTCCTTCGAGCTGTTTGC<br>GCGTGTAAAGTCACCACCCT	*Mus musculus*	qPCR - *PPIA* (housekeeping)	This study
TGACCAACAAGGAGATGCGT<br>AATTGTCCGATTTGCTGCGG	*Homo sapiens*	qPCR - *S1PR1*	This study
GCCTCTCTACGCCAAGCATT<br>GCAGCCAGCAGACGATAAAG	*Homo sapiens*	qPCR - *S1PR2*	This study
TGAAGTCCAGCAGCCGTAAG<br>AGCCAACACGATGAACCACT	*Homo sapiens*	qPCR - *S1PR3*	This study
CATCATGGGCCTCTATGGGG<br>AGAGGTTGGAGCCAAAGACG	*Homo sapiens*	qPCR - *S1PR4*	This study

CTTTGTGGCATGTTGGGGC<br>GCGTGTAGATGATGGGGTTCA	*Homo sapiens*	qPCR - *S1PR5*	This study
GACTGCCTGATTGACAAGCG<br>ATTCTCGTTCCGGTCCTTGC	*Homo sapiens*	qPCR - *RXRA*	This study
ATGCTGGCTATGAGCAGGTC<br>CCCAGACGCCGATACTTCTC	*Homo sapiens*	qPCR - *Sphk1*	This study
CCCCGGTTGCTTCTATTGGT<br>GATCTAGGAGCCCGTTCAGC	*Homo sapiens*	qPCR - *Sphk2*	This study
GACCAAAGCAAAGGCGAGGG<br>CAGCCCTGAAAGATGCGGATG	*Homo sapiens*	qPCR - *PPARG*	This study
GCGAACGATTCGACTCAAGC<br>TCGTCCAAAACGAATCGCGT	*Homo sapiens*	qPCR - *PPARA*	This study
GTCTCCTTTGAGCTGTTTGCAG<br>GGACAAGATGCCAGGACCC	*Homo sapiens*	qPCR - PPIA (housekeeping)	6
GACCTCGCAGATCCAGCAG<br>ATAGGCAGCTTCTCCGCATC	*Homo sapiens*	qPCR - *SREBF1*	7
GTGCTGTTCCTGACTCCCTG<br>CAGCCTTCTTCTTGGCCTGA	*Homo sapiens*	qPCR - *SREBF2*	7
TTCGTTGCCACTTTCTTGCG<br>TGGTGGTAGTTGTGGAAGCC	*Homo sapiens*	qPCR - *SCD*	6
CCATCTGCTTGGTTTCGTGC<br>AGACGGTGTGAAAGAGCCAG	*Homo sapiens*	qPCR - *AdipoR1*	6
TCATCTGTGTGCTGGGCATT<br>CTATCTGCCCTATGGTGGCG	*Homo sapiens*	qPCR - *AdipoR2*	6
TCTCGCAGGTTGTGTCTTCC<br>AGCCTCATGGTAAGCCTTGT	*C. elegans*	qPCR - *tba-1*(housekeeping)	This study
CAGTTGGATGGGTATTCCTCCT<br>TCCATGAGAGGGTGGCTTTG	*C. elegans*	qPCR - *fat-5*	This study
GACCCAGTTCTCGTCTTCCA<br>ATCCGAAATAGTGAGCAGCG	*C. elegans*	qPCR - *fat-6*	This study
GCCGTCTTCTCATTTGCTCTC<br>ACGATGATCACGAGCCCATT	*C. elegans*	qPCR - *fat-7*	This study

SUPPLEMENTARY TABLE 2

siRNA used in this study (some sequences were previously used by the authors).

Target Gene	Catalog	Reference (Supplementary)
AdipoR2	J-007801-10	6
Non-Target	D-001810-10	6
PPARA	J-003434-05	-
PPARG	J-003436-08	-
RXRA	J-003443-10	-
S1PR1	J-003655-06	-
S1PR2	J-003952-12	-
S1PR3	J-005208-06	-
SCD	J-005061-07	-
Sphk1	J-004172-10	-
Sphk2	J-004831-10	-
SREBF1	J-006891-05	7
SREBF2	J-009549-05	7

SUPPLEMENTARY REFERENCES

1. Griffiths, B. *et al.* Sterol regulatory element binding protein-dependent regulation of lipid synthesis supports cell survival and tumor growth. *Cancer Metab* **1**, 3 (2013).
2. Bjursell, M. *et al.* Opposing effects of adiponectin receptors 1 and 2 on energy metabolism. *Diabetes* **56**, 583-593 (2007).
3. Rutkowski, D.T. *et al.* Adaptation to ER stress is mediated by differential stabilities of pro-survival and pro-apoptotic mRNAs and proteins. *PLoS Biol* **4**, e374 (2006).
4. Gomez, J.A. & Rutkowski, D.T. Experimental reconstitution of chronic ER stress in the liver reveals feedback suppression of BiP mRNA expression. *Elife* **5** (2016).
5. Zhang, L. *et al.* A novel role of sphingosine 1-phosphate receptor S1pr1 in mouse thrombopoiesis. *J Exp Med* **209**, 2165-2181 (2012).
6. Devkota, R. *et al.* The adiponectin receptor AdipoR2 and its Caenorhabditis elegans homolog PAQR-2 prevent membrane rigidification by exogenous saturated fatty acids. *PLoS Genet* **13**, e1007004 (2017).
7. Ruiz, M. *et al.* Extensive transcription mis-regulation and membrane defects in AdipoR2-deficient cells challenged with saturated fatty acids. *Biochim Biophys Acta Mol Cell Biol Lipids* **1866**, 158884 (2021).

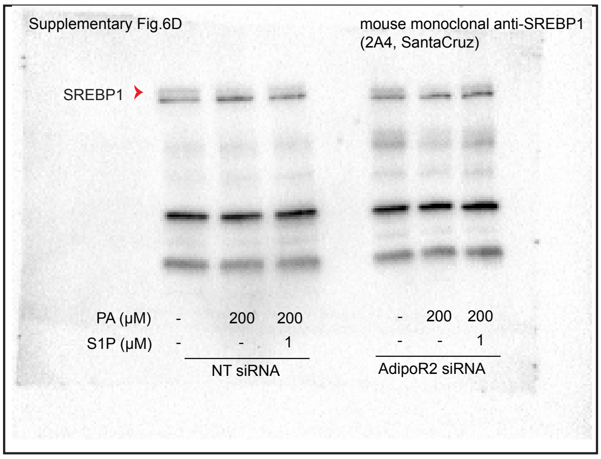
Supplementary Fig.6D
mouse monoclonal anti-SREBP1
(2A4, SantaCruz)
SREBP1
PA (µM) - 200 200 - 200 200
S1P (µM) - - 1 - - 1
NT siRNA
AdipoR2 siRNA

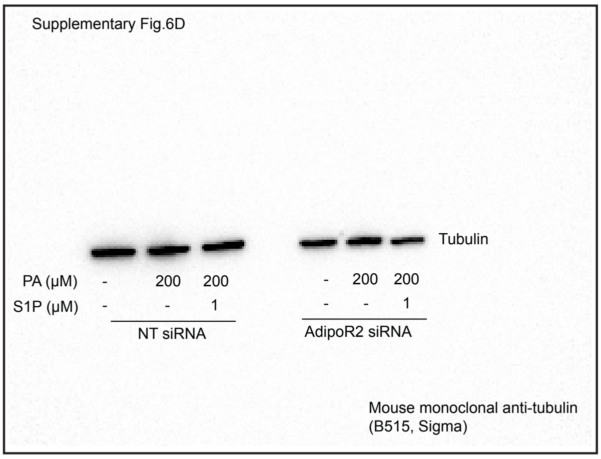
Supplementary Fig.6D
Tubulin
PA (µM) - 200 200 - 200 200
S1P (µM) - - 1 - - 1
NT siRNA
AdipoR2 siRNA
Mouse monoclonal anti-tubulin
(B515, Sigma)
