# Supplementary material for: Sphingosine 1-phosphate mediates adiponectin receptor signaling essential for lipid homeostasis and embryogenesis
Source: Nat Commun. 2022 Nov 22;13:7162. doi: 10.1038/s41467-022-34931-0 (PMC9684441; doi:10.1038/s41467-022-34931-0)
Supplement: Supplementary file 3 — Description of Additional Supplementary Files [file 41467_2022_34931_MOESM3_ESM.pdf]

File name: Supplementary Data 1

Description: Embryo Genotypes

File name: Supplementary Data 2

Description: Embryo Lipidomics

File name: Supplementary Data 3

Description: Embryo Proteomics

File name: Supplementary Data 4

Description: MEFs Lipidomics

File name: Supplementary Data 5

Description: Other Assays

File name: Supplementary Data 6

Description: HEK293 Lipidomics

File name: Supplementary Data 7

Description: C.elegans Lipidomics
