# Supplementary material for: Sphingosine 1-phosphate mediates adiponectin receptor signaling essential for lipid homeostasis and embryogenesis
Source: Nat Commun. 2022 Nov 22;13:7162. doi: 10.1038/s41467-022-34931-0 (PMC9684441; doi:10.1038/s41467-022-34931-0)
Supplement: Supplementary file 5 — Reporting Summary [file 41467_2022_34931_MOESM5_ESM.pdf]

nature portfolio

Corresponding author(s): Mario Ruiz (mario.ruiz.garcia@gu.se)
Marc Pilon (marc.pilon@cmb.gu.se)

Last updated by author(s): Oct 27, 2022

Reporting Summary

Nature Portfolio wishes to improve the reproducibility of the work that we publish. This form provides structure for consistency and transparency in reporting. For further information on Nature Portfolio policies, see our Editorial Policies and the Editorial Policy Checklist.

Statistics

For all statistical analyses, confirm that the following items are present in the figure legend, table legend, main text, or Methods section.

n/a	Confirmed	
☐	☒	The exact sample size (*n*) for each experimental group/condition, given as a discrete number and unit of measurement
☐	☒	A statement on whether measurements were taken from distinct samples or whether the same sample was measured repeatedly
☐	☒	The statistical test(s) used AND whether they are one- or two-sided<br>*Only common tests should be described solely by name; describe more complex techniques in the Methods section.*
☒	☐	A description of all covariates tested
☐	☒	A description of any assumptions or corrections, such as tests of normality and adjustment for multiple comparisons
☐	☒	A full description of the statistical parameters including central tendency (e.g. means) or other basic estimates (e.g. regression coefficient) AND variation (e.g. standard deviation) or associated estimates of uncertainty (e.g. confidence intervals)
☐	☒	For null hypothesis testing, the test statistic (e.g. *F*, *t*, *r*) with confidence intervals, effect sizes, degrees of freedom and *P* value noted<br>*Give P values as exact values whenever suitable.*
☒	☐	For Bayesian analysis, information on the choice of priors and Markov chain Monte Carlo settings
☒	☐	For hierarchical and complex designs, identification of the appropriate level for tests and full reporting of outcomes
☒	☐	Estimates of effect sizes (e.g. Cohen's *d*, Pearson's *r*), indicating how they were calculated

Our web collection on statistics for biologists contains articles on many of the points above.

Software and code

Policy information about availability of computer code

Data collection	No code was used to collect the data
Data analysis	ImageJ 1.47 script: nprot.2011.419-S1 (Reference 68 - Owen et al. Nature Protoc. 2011) - Laurdan Analysis<br>ImageJ 2.1 all other experiments

For manuscripts utilizing custom algorithms or software that are central to the research but not yet described in published literature, software must be made available to editors and reviewers. We strongly encourage code deposition in a community repository (e.g. GitHub). See the Nature Portfolio guidelines for submitting code & software for further information.

Data

Policy information about availability of data

All manuscripts must include a data availability statement. This statement should provide the following information, where applicable:

- Accession codes, unique identifiers, or web links for publicly available datasets
- A description of any restrictions on data availability
- For clinical datasets or third party data, please ensure that the statement adheres to our policy

The proteomics datasets generated during and analysed during the current study are available in the PRIDE repository, [https://www.ebi.ac.uk/pride/]. Project accession: PXD029163.
The lipidomics generated and analyzed during this study are included can be found in the supplementary data and deposit in Zenodo. Project accession: https://zenodo.org/record/7024516#.Y1RRES8RpTY

Field-specific reporting

Please select the one below that is the best fit for your research. If you are not sure, read the appropriate sections before making your selection.

☒ Life sciences ☐ Behavioural & social sciences ☐ Ecological, evolutionary & environmental sciences

For a reference copy of the document with all sections, see nature.com/documents/nr-reporting-summary-flat.pdf

Life sciences study design

All studies must disclose on these points even when the disclosure is negative.

Sample size	Sample sizes and number of replicates were decided prior to each study and based on previous literature using these methods (i.e. Ruiz et al. 2017 37(1):118-129; Devkota et al. 2017 8;13(9):e1007004; Ruiz et al. Elife. 2018 Dec 4;7:e40686 and 2019 Nov 26;8:e47733; Panagaki et al. 2021 118 (30) e2020997118). They are indicated in the figures and exact N numbers can be found in the source data file as well.
Data exclusions	No data were excluded from the analysis.
Replication	All quantitative experiments were performed with a minimum of three independent biological replicates. All quantitative experiments, with the exception of proteomics and lipidomics experiments, were also performed at least twice with similar results.
Randomization	Almost all experiments relied on instrument-based quantification or straightforward scoring of an observable feature in cells, worms or histology sections. For all experiments, individual samples were randomly assigned to different groups/treatments.
Blinding	Almost all experiments relied on instrument-based quantification or straightforward scoring of an observable feature in cells, worms or histology sections. Investigators were therefore not deliberately blinded to group allocation during most data collection. For some analyses, including Laurdan GP quantification, proteomics analysis and lipidomics analysis, samples were handled and analyzed in a blinded fashion because the samples were prepared and cryptically labeled by one individual, then handed to another for analysis.

Reporting for specific materials, systems and methods

We require information from authors about some types of materials, experimental systems and methods used in many studies. Here, indicate whether each material, system or method listed is relevant to your study. If you are not sure if a list item applies to your research, read the appropriate section before selecting a response.

Materials & experimental systems

n/a	Involved in the study	
☐	☒	Antibodies
☐	☒	Eukaryotic cell lines
☒	☐	Palaeontology and archaeology
☐	☒	Animals and other organisms
☒	☐	Human research participants
☒	☐	Clinical data
☒	☐	Dual use research of concern

Methods

n/a	Involved in the study	
☒	☐	ChIP-seq
☐	☒	Flow cytometry
☒	☐	MRI-based neuroimaging

Antibodies

Antibodies used	rabbit monoclonal anti-HA antibody (C29F4, Cell signaling, catalog#14031) 1:5000 dilution, rabbit monoclonal anti-SCD (C12H5, Cell Signaling, catalog#2794) 1:1000 dilution, mouse monoclonal anti-SREBP1 (2A4, Santa Cruz, catalog#sc-13551) 1:200 or mouse monoclonal anti-Tubulin (B512, Sigma, catalog#5168), swine anti-rabbit HRP (1:3000, Dako, catalog#P0399) and goat anti-mouse HRP (1:3000, Dako, catalog#P0447).
Validation	Validation (as detailed in manufacturer's webpage).<br>- C29F4: HA-Tag (C29F4) Rabbit mAb detects exogenously expressed proteins containing the HA epitope tag. The antibody may cross-react with a protein of unknown origin ~100kDa.<br>Species Reactivity: All expected.<br>- C12H5: Rabbit mAb detects endogenous levels of total SCD1 protein. Species cross-reactivity for IHC-P is mouse only.<br>Species Reactivity: Human, Mouse<br>- 2A4: Species Detection/Reactivity: mouse, rat, human, hamster. Additional Mammalian Reactivity: n/a.<br>- B512: Specificity:Recognizes an epitope located at the C-terminal end of the α-tubulin isoform in a variety of organisms.

Eukaryotic cell lines

Policy information about cell lines

Cell line source(s)	HEK293 was purchased from ATCC. Other cell lines were donated from other laboratories: INS-1E (Dr. V. Sukonina and Prof. S. Enerbäck, University of Gothenburg, Sweden), JURKAT E6.1 (Prof. L. Nilsson and Prof. J. Nilsson, University of Gothenburg, Sweden), U-2 OS (Dr. A. Besse and Dr. L. Besse, Cantonal Hospital St. Gallen, Switzerland).
Authentication	HEK293 were authenticated by STR profiling in 2018, before the start of the study. The other lines were not tested.
Mycoplasma contamination	Mycoplasma abscence was verified in HEK293 before the start of the study. The other lines were not tested.
Commonly misidentified lines (See ICLAC register)	None

Animals and other organisms

Policy information about studies involving animals; ARRIVE guidelines recommended for reporting animal research

Laboratory animals	Mus musculus, C57Bl/6, embryos at E12.5 and E15.5.<br><br>C.elegans, larva and young adults (less than a week).<br>The wild-type C. elegans reference strain N2, paqr-2(tm3410), sphk-1(ok1097), nhr-49(gk405) and sbp-1(ep79) and the transgene carrying strain EG7865 {oxTi617 [eft-3p::tdTomato::H2B::unc-54 3'UTR + Cbr-unc-119(+)]} are available from the C. elegans Genetics Center (CGC; USA). The pfat-7::GFP(rtIs30) carrying strain HA1842 was a kind gift from Amy Walker.
Wild animals	The study did not involved wild-animals.
Field-collected samples	The study did not involved samples collected from the field.
Ethics oversight	Ethical permit (#2311/19) approved by the Gothenburg Animal Ethics Committee

Note that full information on the approval of the study protocol must also be provided in the manuscript.

Flow Cytometry

Plots

Confirm that:

☐ The axis labels state the marker and fluorochrome used (e.g. CD4-FITC).

☐ The axis scales are clearly visible. Include numbers along axes only for bottom left plot of group (a 'group' is an analysis of identical markers).

☐ All plots are contour plots with outliers or pseudocolor plots.

☐ A numerical value for number of cells or percentage (with statistics) is provided.

Methodology

Sample preparation	Live cells were stained with Laurdan dye (6-dodecanoyl-2-dimethylaminonaphthalene) (Thermo Scientific) at 15 µM for 45 min (in HBSS without Ca2+ and Mg2+ at pH 7.4 supplemented with 10 mM HEPES).
Instrument	Cytoflex S (Beckman Coulter)
Software	Kaluza software (Beckman Coulter) version 2.1
Cell population abundance	All the cells were included in the analysis. 100%.
Gating strategy	No gating strategy was used. All the cells were considered positive, namely stained with Laurdan - and included in the analysis. Thus, no figure exemplifying the gating is included in the manuscript.

☐ Tick this box to confirm that a figure exemplifying the gating strategy is provided in the Supplementary Information.
