# Supplementary figures and images for: Sphingosine 1-phosphate mediates adiponectin receptor signaling essential for lipid homeostasis and embryogenesis
Source: Nat Commun. 2022 Nov 22;13:7162. doi: 10.1038/s41467-022-34931-0 (PMC9684441; doi:10.1038/s41467-022-34931-0)

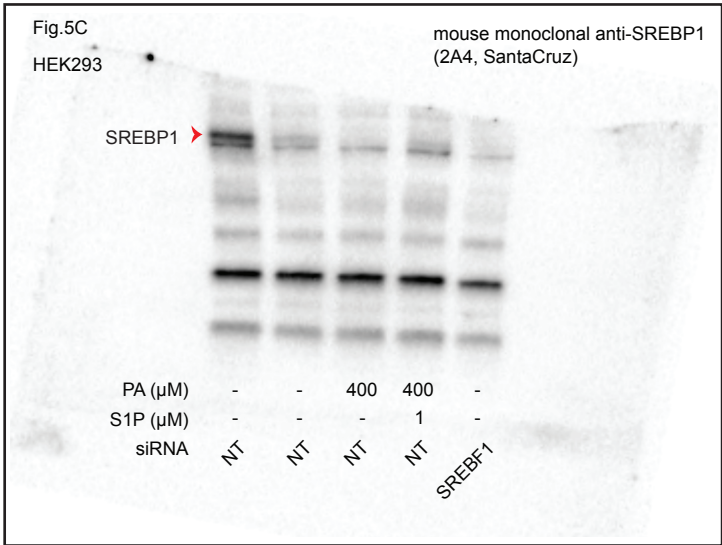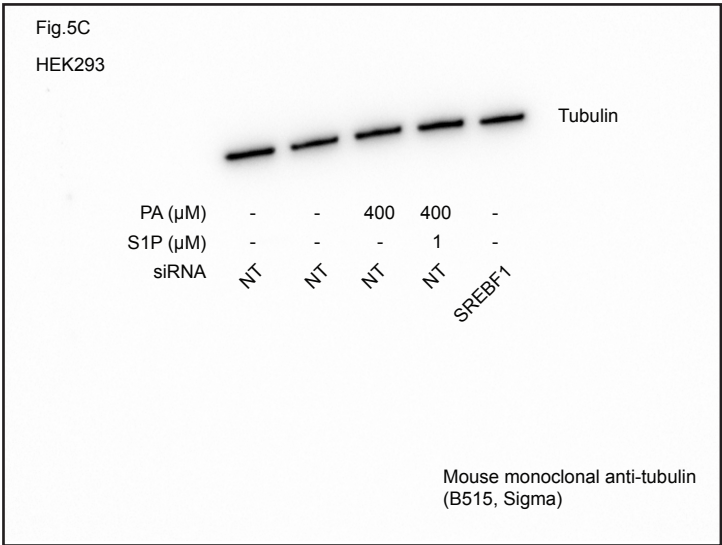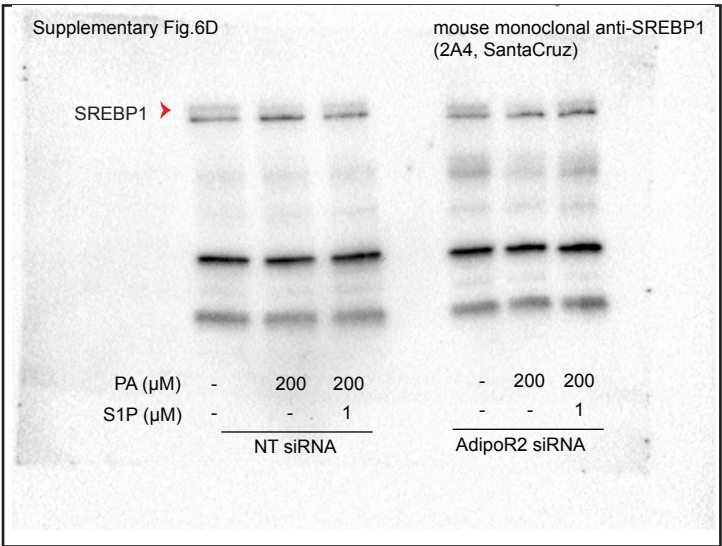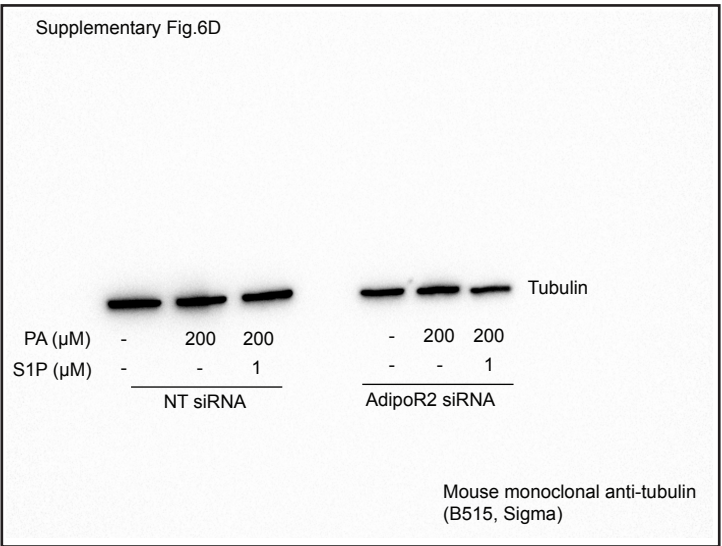

Supplement: Supplementary file 6 — Source Data [file 41467_2022_34931_MOESM6_ESM.zip › Source Data NCOMMS-21-38753A/Source Data WB 2.pdf]

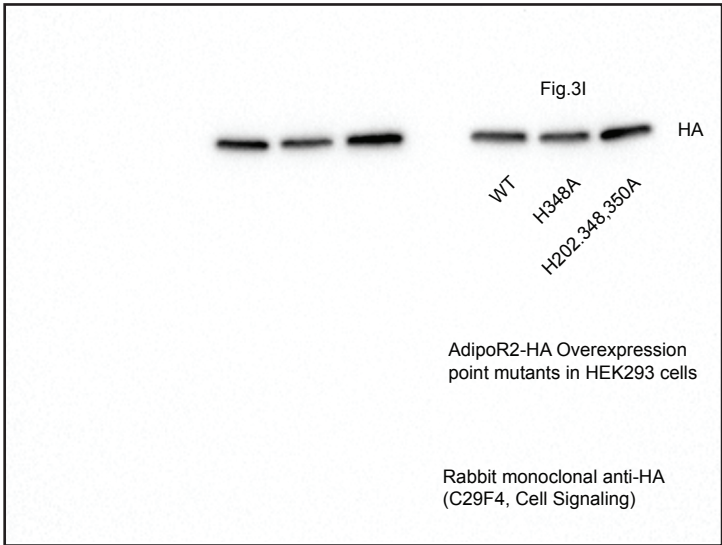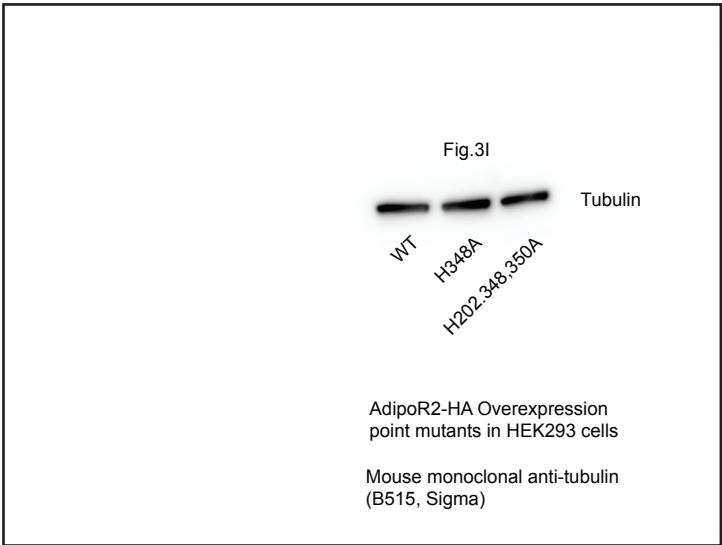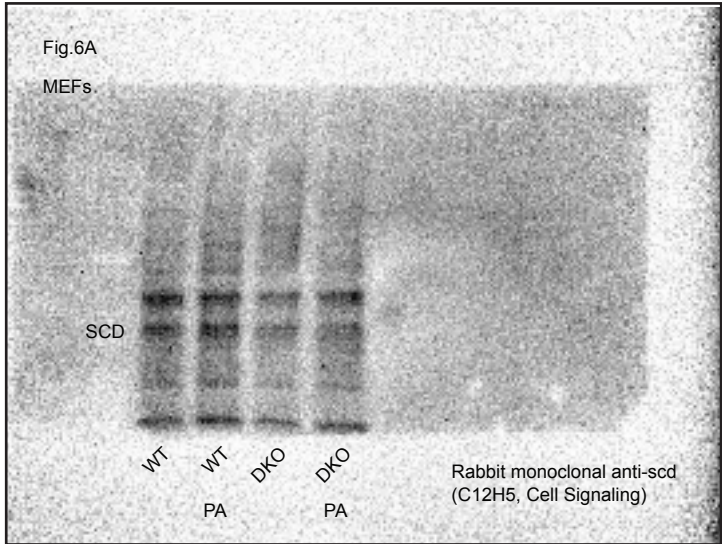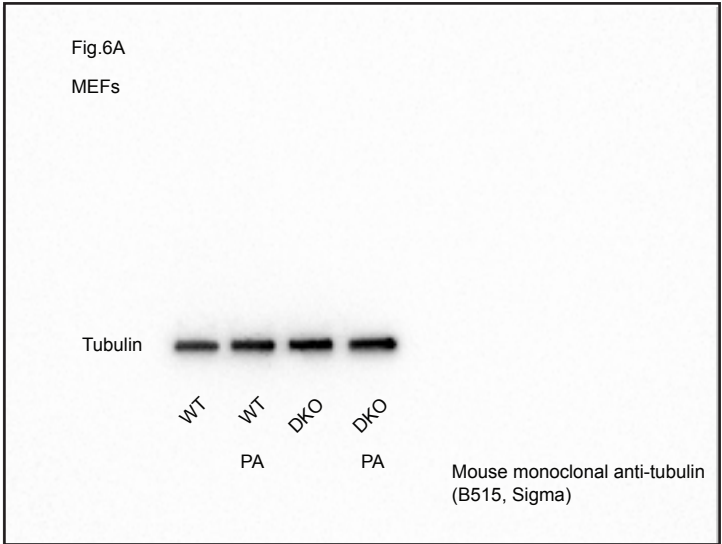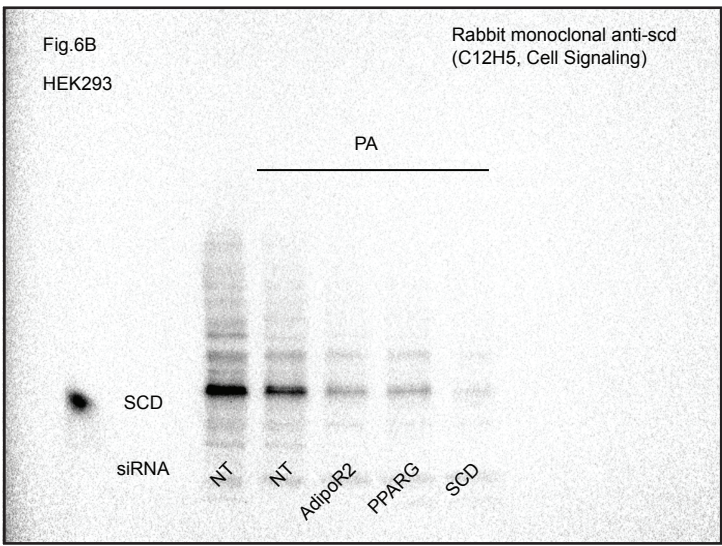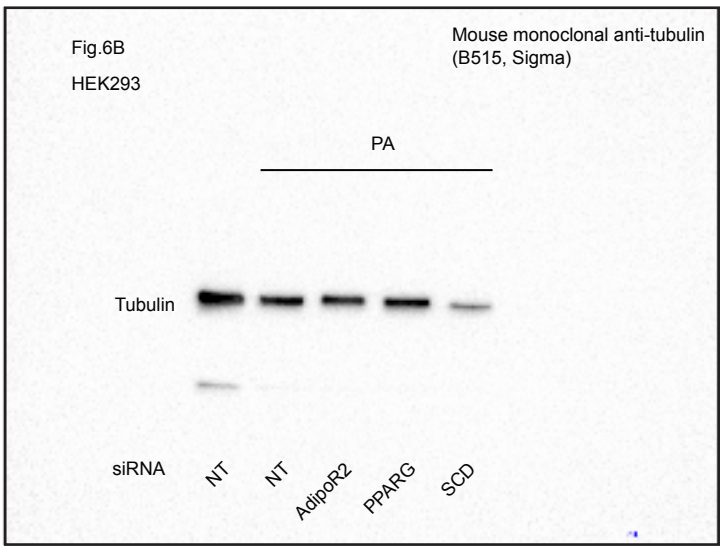

Supplement: Supplementary file 6 — Source Data [file 41467_2022_34931_MOESM6_ESM.zip › Source Data NCOMMS-21-38753A/Source Data WB 1.pdf]
